# The impact of 24-h activity patterns on executive function in older adults with chronic diseases: analysis of the isotonic substitution effect

**DOI:** 10.3389/fpubh.2026.1733294

**Published:** 2026-02-23

**Authors:** Zhiji Wang, Lin Wang, Shijie Liu, Li Zhan, Sijun Wu, Linxia Tang, Hong Wang

**Affiliations:** 1 School of Physical Education, Wuhan University of Technology, Wuhan, Hubei, China; 2 Center for Hubei Ethnic Traditional Sports Culture Preservation and Innovation, Wuhan, Hubei, China; 3 Shanghai University of Sport, Shanghai, China; 4 Department of Physical Education, Shanghai University of Traditional Chinese Medicine, Shanghai, China; 5 Wushu College, Wuhan Sport University, Wuhan, Hubei, China

**Keywords:** 24-h activity behaviors, chronic disease, component data analysis, executive function, older adult

## Abstract

**Objective:**

24-h activity encompasses four categories: light-intensity physical activity (LPA), moderate-to-vigorous-intensity physical activity (MVPA), sedentary behavior (SB), and sleep (SP). This study aims to investigate the effects of different physical activity components on executive function in older adults with chronic diseases and to examine substitution effects among activity components. The findings provide scientific evidence to inform physical activity interventions for improving executive function in older adults with chronic diseases.

**Methods:**

A total of 105 older adults (72.64 ± 6.82 years) were recruited. Following questionnaire screening, 75 older adults with chronic diseases were ultimately included. Accelerometers objectively measured participants’ daily SP, SB, LPA, and MVPA. Executive function was objectively assessed using the Stroop task, N-back task, and More-odd-shifting task. Component linear regression equation assessed the relationship between different activities and executive function in older adults with chronic diseases. The dose–response effects of “one-for-one” substitutions between different activity behaviors were explored.

**Results:**

Component linear regression results showed that SB positively correlated with inhibitory control (*β* = 0.892, 95% CI = 0.044 to 1.741) and working memory (*β* = 1.610, 95% CI = 0.801 to 2.420). MVPA was negatively correlated with inhibitory control (*β* = −0.596, 95% CI = −1.156 to −0.036), while LPA was negatively correlated with working memory (*β* = −0.969, 95% CI = −1.812 to −0.127). No significant association with cognitive flexibility was found (*p* > 0.05). Isotemporal substitution results showed that for inhibitory control, replacing SB with SP for 30 min reduced z-scores by 0.068; replacing SB with MVPA for 30 min reduced z-scores by 0.285. For working memory, replacing SB with LPA every 30 min resulted in a z-score decrease of 0.152. Dose–response curves indicated that progressively increasing the proportion of time spent in SP and MVPA improved inhibitory control, while increasing LPA enhanced working memory.

**Conclusion:**

SP and MVPA significantly improve inhibitory control in older adults with chronic diseases, while LPA significantly enhances their working memory. It is recommended that older adults with chronic diseases adjust their daily time structure by increasing diverse physical activities, ensuring adequate sleep duration, and reducing sedentary behavior to improve executive function.

## 1 Introduction

Older adults, affected by factors such as declining physical function and weakened immunity, constitute the demographic with the highest prevalence and incidence rates of chronic diseases (1). Data released by China’s National Health Commission shows that nearly 180 million Chinese older adults suffer from chronic diseases, accounting for 67.4% of the total older population (2). Existing research indicates that older patients with chronic diseases face severe challenges including physical decline, cognitive deterioration, increased financial burdens, and heightened mortality risks. Among these, common chronic conditions such as hypertension, hyperlipidemia, and diabetes exhibit a high correlation with cognitive impairment and dementia incidence in older adults (3). Executive functions encompass three core components: inhibitory control, working memory, and cognitive flexibility. They form the foundation for reasoning, problem-solving, and planning abilities, and represent the cognitive functions most susceptible to age-related changes in brain structure and function. Their performance is linked to brain activity patterns and directly reflects an individual’s proficiency in motor control (4, 5). The better the executive function in older adults, the higher their motivation to participate in physical activities, and the better their balance control and ability to prevent falls (6). Therefore, how to effectively promote healthy aging among older adults with chronic diseases and effectively prevent common mental disorders and behavioral problems such as Alzheimer’s disease are pressing challenges that public health initiatives must address to advance healthy aging.

Existing research suggests that daily activity behaviors such as physical activity, sedentary behavior, and sleep are broadly associated with cognitive function in older adults (7). A meta-analysis indicates that regular physical exercise, particularly aerobic and mind–body exercises, offers positive benefits for improving working memory, cognitive flexibility, and inhibitory control in older adults with cognitive health (8). Sedentary behavior, in contrast to daily activity patterns, is associated with reduced health outcomes. Decreasing sedentary time and increasing physical activity duration are linked to favorable health indicators. Individuals sleeping 6–8 h per night exhibit greater gray matter volume in frontal, temporal, parietal, and cerebellar regions, with optimal sleep duration per hour correlating to differences in cognitive performance (9). A cross-sectional study indicates that older adults with daily sedentary periods exceeding 8 h, compared to those below 8 h, exhibit lower hippocampal volume, leading to memory impairment and increased dementia risk (10). For older adults with chronic diseases, a study using commercial wearable devices shows that sleep patterns (stages, duration, and regularity) correlate with chronic disease incidence (11). The same study further demonstrated that replacing sedentary behavior with physical activity of varying intensities is associated with reduced risks for 45 common non-communicable diseases. Substituting sedentary time with light, moderate, or vigorous physical activity all positively contributes to lowering the risk of adverse health outcomes (12). In summary, daily activity patterns represent a crucial modifiable factor influencing cognitive function in older adults, particularly in delaying memory decline and reducing chronic disease risks.

Since a day invariably consists of 24 h, it is composed of light-intensity physical activity (LPA), moderate-to-vigorous-intensity physical activity (MVPA), sedentary behavior (SB), and sleep (SP). When the duration of one physical activity component changes, it inevitably leads to changes in the duration of the other physical activity components (13). Traditional epidemiological studies often use linear regression to examine the relationship between a single component of daily activity and executive function, thereby overlooking the interconnections among 24-h activity (14). However, when combining various components of daily activity, the issue of multicollinearity among these activity components cannot be ignored (15). Component data analysis (CoDA) effectively addresses multicollinearity issues among component data. Since Chastin et al. (16) introduced CoDA in 2015 as a technique for analyzing daily time use, researchers have attempted to apply this analytical method to examine 24-h activity patterns and health outcomes in older adults (16–18). Compared to single-behavior interventions, integrated 24-h behavioral pattern management that incorporates physical activity demonstrates significant advantages. It is more suitable for older adults with varying health foundations and helps promote the development of executive function indicators in older populations with chronic diseases. However, these studies primarily rely on questionnaires to quantify executive function in older adults, lacking objective quantitative tools. Furthermore, few scholars have examined the impact of various 24-h activities on executive function in older adults with chronic diseases.

Given the previous lack of research examining the relationship between 24-h activity patterns and executive function in older adults with chronic diseases, we employed component data analysis to investigate whether 24-h activity patterns correlate with executive function performance in this population. We further explored how temporal reallocation among 24-h activities relates to executive function performance. This study contributes to enriching the theoretical foundation regarding the relationship between 24-h behavior and executive function in older adults with chronic diseases. It provides scientific evidence for improving executive function in this population and developing effective intervention strategies tailored to their needs.

## 2 Materials and methods

### 2.1 Participants

This study employed a cross-sectional survey design. Sample size was calculated using GPower Win 3.1.9.7, assuming an effect size f^2^ = 0.15, a significance level *α* = 0.05, and (1-*β* err prob) = 0.85. The final estimated minimum sample size was 63 participants. Recruitment notices were posted in September 2024 at the East and West campuses of Wuhan University of Technology, resulting in 112 participants enrolled in the study. Older adults with chronic diseases were defined as those suffering from at least one chronic condition, screened via a health status questionnaire. Chronic disease categories included: no chronic disease; cardiovascular system diseases (hypertension, heart disease, cerebrovascular disease, dyslipidemia); diabetes; chronic lung disease; digestive system diseases (gastric disease, liver disease, gallbladder or spleen disease); kidney disease; bone disease; mental illness; malignant tumors; oral disease; and eye disease. Inclusion criteria were: (1) age ≥60 years; (2) ability to independently complete computer-based testing tasks; (3) presence of at least one chronic disease. Exclusion criteria were: (1) severe cognitive impairment (e.g., professionally diagnosed dementia); (2) hearing, visual, or communication impairments; (3) missing 24-h activity data. This study was approved by the Scientific Research Ethics Committee of Wuhan Sport University (Approval no.: 2025119). All participants voluntarily enrolled and signed informed consent forms.

### 2.2 Executive function

All executive function tests were administered using E-prime 2.0.10.182 software on computers. During the experiment, researchers guided participants into a quiet, isolated environment to sequentially complete the executive function task tests. Before each task began, researchers uniformly explained the operational requirements to ensure standardized procedures. To compare the relative effects of physical activity on inhibitory control (Stroop), working memory (N-back), and cognitive flexibility (More-odd shifting), the dependent variables for each cognitive task were converted into task-standardized z-scores.

#### 2.2.1 Stroop task

The Stroop task, a measure of inhibitory control that is both simple and easily implementable, is the most commonly utilized paradigm for the study of executive functions (19). In this task, Chinese characters (e.g., “红,” “黄,” “蓝,” “绿”) are displayed on a computer screen alongside their respective colours (i.e., red, yellow, blue and green). If the characters and colours match, the participant is instructed to respond promptly by pressing the “F” button, while if they do not match, the participant is instructed to press the “J” button. The word-colour task commences with a 500-millisecond gaze point “+” positioned centrally on the screen, followed by a 1,000-millisecond interval during which the stimulus picture is displayed for a duration of 1,500 milliseconds. During this interval, the participant is permitted to press the designated key. Subsequently, the next set of word colours is presented. The Stroop task consists of 16 practice trials and 72 formal trials. The correct response time for trials that are inconsistent is recorded as the dependent variable.

#### 2.2.2 N-back task

The measurement of working memory was conducted through the utilization of the N-back task (20). In this experiment, the results of the 1-back test were employed to evaluate the executive functioning of older adults, with Arabic numerals serving as the stimulus material. In the 1-back task, a single number is presented randomly, and the subject is tasked with memorizing the number. Subsequently, upon the appearance of the subsequent number, the subject is prompted to compare it with the initially presented number. Reactions were recorded as either “F” for identical numbers or “J” for different numbers. The 1-back task commenced with the presentation of the gaze point “+” for 1,000 milliseconds, followed by the presentation of the stimulus digit for 1,500 milliseconds. Thereafter, the subject was permitted to press a key for a rapid response, and the subsequent empty screen was presented for 1,000 milliseconds prior to the presentation of the next gaze point. This task phase encompassed 12 practice sessions and 120 sessions of the formal experiment. The formal experiment phase comprised one round every 40 times, followed by a rest interval. The subsequent round of testing was initiated upon the pressing of any key by the subject. The accurate response time for the 1-back task was documented as the dependent variable.

#### 2.2.3 More-odd shifting task

The More-odd Shifting task is a widely utilized tool for the assessment of cognitive flexibility (21). The task is composed of three distinct sections: size judgement, parity judgement, and mixed judgement. In the size judgement section, the red numbers “1–9” are presented, with “5” being absent. Subjects are required to ascertain the numerical value of the red numbers and the numerical value of “5.” If “5” is smaller than the red number, the subject is required to press the “F” key. If “5” is larger than the red number, the subject is required to press the “J” key. In the parity judgement section, green numbers “1–9” are presented. Subjects are required to make a parity judgement on the numerical values of the green numbers. If the numerical values of the green numbers are odd, the subject is required to press the “F” key. If the numerical values of the green numbers are even, the subject is required to press the “J” key. Red and green numbers will appear alternately in the centre of the screen in the mixing section, and subjects will use the size judgement rule to make a judgement if a red number appears, or the parity judgement rule to make a judgement if it is an even number, and respond. The time when the correct response was recorded for the mixed section was used as the dependent variable.

#### 2.2.4 24-h movement behaviors

The measurement of physical activity data for subjects was conducted using the ActiGraph wGT3X-BT accelerometer, a device that has been tested for its ability to objectively and accurately quantify daily physical activity, sedentariness, and sleep, while also providing complete and continuous activity data (22). The accelerometer data collection time unit (epoch) was set at 60 s, and the measurement duration was a full and continuous 7 days (comprising 5 working days and 2 rest days), with daily wear time ≥ 10 h recorded as valid data. Participants who wore the device for ≥ 3 days (2 working days and 1 rest day) were included in the analyses. Subjects were asked to wear the sensors on the wrist of their non-dominant hand during the test period and were asked to keep them on at all times except when necessary (e.g., bathing, swimming, etc.). Data collection began at 12:00 on the day after distribution and ended at 12:00 on the eighth day, after which the apparatus was recovered by the experimenter. Physical activity behaviors were classified according to the calculation method of physical activity time for each intensity proposed by Miller et al. (61) 0–99 counts/min was recorded as SB, 100–1951 counts/min was recorded as LPA, and more than 1951 counts/min was recorded as MVPA, and the data were exported through Actilife 6.13.3 (23). Furthermore, the physical activity of the subjects at each time period was also collected by means of a paper physical activity log. This log included the start and end times of the subjects’ activity behavior and sleep at each time period. The content of this log will be used as a subjective record of the subjects to assist the researcher in calibrating the physical activity data.

#### 2.2.5 Covariates

Information of a sociodemographic nature was collected in the form of a paper questionnaire, including age, gender, height, weight, and educational attainment (primary school or lower, secondary school, bachelor’s degree and above).

#### 2.2.6 Statistical analysis

All mathematical statistics were performed in R 4.5.0, following the guidelines for 24 h activity analysis (CoDA) proposed by Dumuid et al. (14) using the “compositions” package and the “robCompositions” package statistical analyses (14). In order to avoid the problem of “fixed-sum limitations and covariances” between the component data from interfering with the analysis results, a variance matrix was used to reflect the discrete trends and interdependencies between the component data (24). Subsequently, the equidistant logarithmic ratio method was used to convert the physical activity data into ilr coordinates of a group of three data, which was mapped from a monomorphic space to a Euclidean space to eliminate the problem of multicollinearity among the component data. After establishing the four sets of coordinates for SP, SB, LPA, and MVPA respectively, the component multiple linear regression models were established by adjusting gender, age, and education level as covariates, and the associations between each activity behavior and the executive function of chronically ill older adults were examined separately with different executive function test results as the dependent variables. Following the confirmation of a statistically significant association between time spent in physical activity and executive functioning indicators, time-component isochronous substitution was performed. This involved the assessment of the change in executive functioning indicators based on the reassignment of the paired physical activity compositions (25). This statistical method takes the mean of participants’ physical activity time compositions as the baseline value for physical activity data and uses this to perform pairwise time substitution. The new compositions were then used to predict changes in executive function metrics, and the difference between the values obtained from the new compositions and the original values was considered to be the theoretical change in executive function when the two behaviors were reallocated. In the present study, the temporal sequence of behavior was modified in 30-min increments (for example, reallocating 30 min of SP to SB), while maintaining constant the remaining behavioral time shares. Subsequent exploration and plotting of dose-effect relationships between behaviors was conducted in 5-min increments up to the mean of the least behavioral composition (16).

## 3 Results

### 3.1 Participant characteristics

Subjects were screened based on information about chronic diseases, and finally, 75 older adults with chronic diseases were included (Table 1). The participants’ mean age was 72.64 ± 6.82 years. Eighteen (24%) were male and 57 (76%) were female. Their mean height was 161.80 ± 6.68 cm and their mean body weight was 61.03 ± 8.67 kg. The chronic disease information showed that cardiovascular diseases were the most prevalent, affecting 80% of the total participants (*N =* 60). Digestive system diseases (*n =* 37), bone diseases (*n =* 37), eye diseases (*n =* 32), and oral diseases (*n =* 26) followed. Fewer participants had diabetes (*n =* 9), chronic lung disease (*n =* 6), kidney disease (*n =* 5), mental illness (*n =* 4), or malignancy (*n =* 2). According to the 24-h activity behavior data of chronically ill older adults, the geometric mean values of SB, LPA, and MVPA were 527.35, 703.97, and 44.30 min, respectively. In the executive function index, the mean response times were 1,109.63 ms for the Stroop test, 541.26 ms for the 1-back test, and 788.67 ms for the odd-shifting test.

**Table 1:** Baseline characteristics of participants.

Variable	Total (*n =* 75)	Male (*n =* 18)	Female (*n =* 57)
Age (years)	72.64 ± 6.82	74.50 ± 6.99	72.05 ± 6.72
Height (cm)	161.80 ± 6.68	169.61 ± 5.15	159.33 ± 5.01
Weight (kg)	61.03 ± 8.67	67.78 ± 8.51	58.90 ± 7.62
Education level			
Elementary school or below, *N* (%)	9 (12.0)	0 (0.0)	9 (15.8)
Middle school, *N* (%)	30 (40.0)	3 (17.0)	27 (47.4)
Bachelor’s degree or higher, *N* (%)	36 (48.0)	15 (83.0)	21 (36.8)
Executive function			
Inhibitory Control (ms)	1109.63 ± 140.31	1095.15 ± 136.57	1114.20 ± 142.35
Working Memory (ms)	541.26 ± 73.81	551.58 ± 80.26	538.00 ± 72.10
Cognitive Flexibility (ms)	788.67 ± 75.59	811.20 ± 87.71	781.55 ± 70.71
Physical activity			
SP (min)	527.35 (36.6)	529.94 (36.8)	526.40 (36.6)
SB (min)	703.97 (48.9)	685.22 (47.6)	709.82 (49.3)
LPA (min)	164.39 (11.4)	176.32 (12.2)	160.75 (11.1)
MVPA (min)	44.30 (3.0)	48.52 (3.3)	43.04 (3.0)
Cardiovascular Disease, *N* (%)	60 (80.0)	12 (66.7)	48 (84.2)
Diabetes, *N* (%)	9 (12.0)	3 (16.7)	6 (10.5)
Chronic lung disease, *N* (%)	6 (8.0)	0 (0.0)	6 (10.5)
Digestive System Diseases, *N* (%)	37 (49.3)	8 (44.4)	29 (50.9)
Kidney disease, *N* (%)	5 (6.7)	3 (16.7)	2 (3.5)
Bone disease, *N* (%)	37 (49.3)	7 (38.9)	30 (52.6)
Mental disorders, *N* (%)	4 (5.3)	0 (0.0)	4 (7.0)
Malignant tumors, *N* (%)	2 (2.7)	1 (5.6)	1 (1.8)
Oral diseases, *N* (%)	26 (34.7)	8 (44.4)	18 (31.6)
Eye disease, *N* (%)	32 (42.7)	7 (38.9)	25 (43.9)

### 3.2 Variability of physical activity

By constructing the variance matrix of components (Table 2), we examined the variability across the entire sample activity dataset to reveal dependencies among variables within the component data. The closer the ratio of two behavioral components is to zero, the stronger the temporal interdependence between these behaviors. The isometric logarithmic ratio (lnSB/SP = 0.051) exhibits the lowest variability, indicating that SB is most strongly correlated with standing behavior (SP) and most readily convertible between the two. Compared to the other three behaviors, MVPA exhibits greater variability in its logarithmic ratios (lnMVPA/SP = 0.148; lnMVPA/SB = 0.214; lnMVPA/LPA = 0.143). This indicates that MVPA possesses the weakest temporal dependency relative to the other three activity behaviors.

**Table 2:** Variation matrix of physical activity components.

Component	SP	SB	LPA	MVPA
SP	0	0.051	0.085	0.148
SB	0.051	0	0.065	0.214
LPA	0.085	0.065	0	0.143
MVPA	0.148	0.214	0.143	0

### 3.3 Compositional data regression analysis

Following the adjustment for gender, age, and education, regression equations were constructed to investigate the relationships between distinct physical activities and executive functioning. The transformed ilr coordinates of SP, SB, LPA, and MVPA were designated as independent variables (Table 3). The comprehensive evaluation of the model revealed statistically significant associations of 24-h activity behavioral composition with both inhibitory function (*p <* 0.05) and working memory (*p <* 0.05). However, the association with cognitive flexibility did not attain the level of statistical significance (*p* > 0.05). With respect to the relative contribution of different activity behaviors, the analysis found that SB was significantly and positively associated with inhibitory control (*β* = 0.892, 95% CI = 0.044 to 1.741) and working memory (*β* = 1.610, 95% CI = 0.801 to 2.420). This finding indicates that with each additional unit of SB, there is a concomitant decline in inhibitory control and working memory performance. In contrast, the magnitude of the correlation between MVPA and inhibitory control was found to be significantly negative (*β* = −0.596, 95% CI = −1.156 to −0.036). Similarly, the correlation between LPA and working memory was found to be significantly negative (*β* = −0.969, 95% CI = −1.812 to −0.127). This finding indicates that an increase in MVPA per unit is associated with enhanced inhibitory control. Conversely, an augmentation in LPA levels has been demonstrated to correlate with enhanced working memory. Moreover, the findings revealed that none of the observed associations between SP and the selected executive function indicators attained statistical significance.

**Table 3:** Linear regression results for components of 24-h activity behavior and executive function in older adults with chronic diseases.

Activity behavior	Inhibitory control	Working memory	Cognitive flexibility
	*β* (95% CI)	*β* (95% CI)	*β* (95% CI)
SP	0.064 (−0.967, 1.097)	0.715 (−0.267, 1.697)	−0.267 (−1.367, 0.835)
SB	0.892* (−0.044, 1.741)	1.610* (0.801, 2.420)	0.120 (−0.770, 1.011)
LPA	0.139 (−0.725, 1.002)	−0.969*(−1.812, −0.127)	−0.437 (−1.351, 0.477)
MVPA	−0.596*(−1.156, −0.036)	0.069 (−0.476, 0.615)	0.266 (−0.331, 0.862)
Model P	*p <* 0.05	*p <* 0.05	*p* > 0.05

*Indicates significance level <0.05.

### 3.4 Reallocating of 24-h activity duration and its impact on executive function prediction with older adults with chronic diseases

The substitution benefits of different physical activities on inhibitory function and working memory were explored after substituting each physical activity for 30 min (Table 4). The predicted results indicated that replacing SP and MVPA with 30 min of SB resulted in alterations in inhibitory function of 0.068 (95% CI = 0.004 to 0.133) and 0.578 (95% CI = 0.046 to 1.109), respectively, in older adults with chronic disease. In contrast, the replacement of SB with 30 min LPA or SP resulted in a predicted outcome change of −0.068 (95% CI = −0.131 to −0.005) and −0.285 (95% CI = −0.525 to −0.046), respectively. In other words, a reduction in 30-min MVPA or SB was associated with diminished inhibitory function performance. In working memory, the 30-min LPA replacement for SB exhibited a predicted change value of −0.152 (95% CI = −0.266 to −0.037), while the SB replacement for LPA predicted a change value of 0.175 (95% CI = 0.039 to 0.311). These findings suggest that substituting the 30-min LPA for SB is associated with enhanced working memory.

**Table 4:** Expected changes in executive function dimensions following 24-h activity-based isotemporal substitution.

Replacing/replaced activity	Inhibitory control (95% CI)	Working memory (95% CI)
SP/SB	−0.068* (−0.131, −0.005)	−0.035 (−0.096, 0.026)
SP/LPA	−0.065 (−0.220, 0.091)	0.142 (−0.010, 0.293)
SP/MVPA	0.511 (−0.041, 1.064)	−0.069 (−0.606, 0.468)
SB/SP	0.068* (0.004, 0.133)	0.034 (−0.029, 0.096)
SB/LPA	0.002 (−0.138, 0.141)	0.175* (0.039, 0.311)
SB/MVPA	0.578* (0.046, 1.109)	−0.036 (−0.552, 0.481)
LPA/SP	0.061 (−0.075, 0.196)	−0.117 (−0.248, 0.015)
LPA/SB	−0.009 (−0.127, 0.109)	−0.152* (−0.266, −0.037)
LPA/MVPA	0.570 (−0.046, 1.186)	−0.186 (−0.785, 0.412)
MVPA/SP	−0.216 (−0.479, 0.048)	0.034 (−0.222, 0.289)
MVPA/SB	−0.285* (−0.525, −0.046)	−0.001 (−0.234, 0.232)
MVPA/LPA	−0.282 (−0.630, 0.066)	0.175 (−0.163, 0.513)

Cognitive flexibility significance levels >0.05 are not shown in this table; *indicates significance level <0.05.

### 3.5 Dose-response relationship between 24-h activity patterns and executive function in older adults with chronic diseases

**Figure 1:**
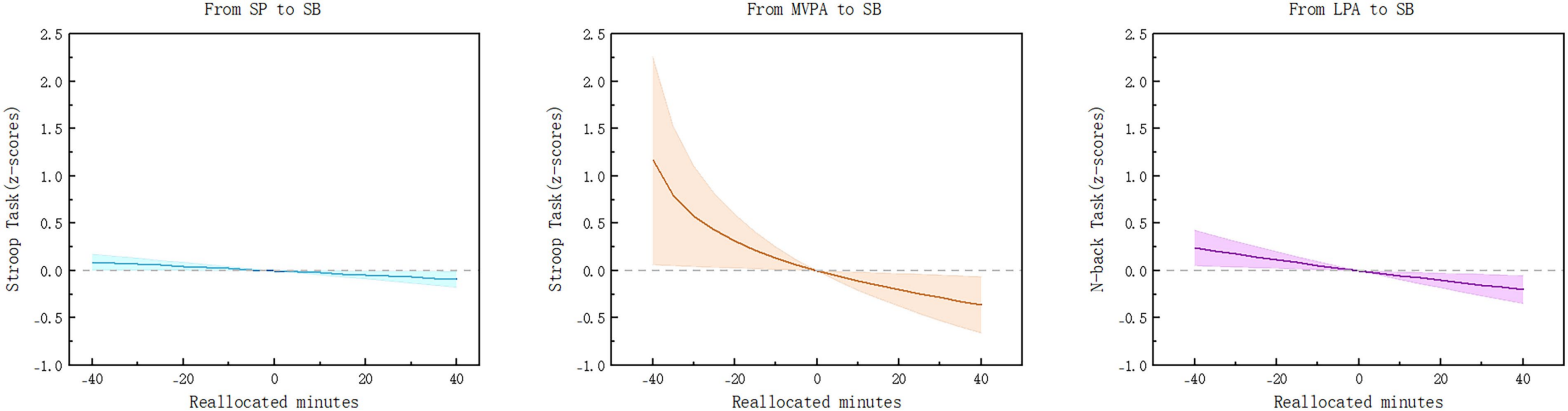
Dose-response relationship between 24-h activity patterns and executive function in older adults with chronic diseases.

To further reveal the dose-effect relationship of isochronous changes in physical activity on executive function in older adults with chronic diseases, we plotted the trend of the effect of isochronous substitution on executive function between two behaviors in 5-min increments (Figure 1). In this study, the arithmetic mean value of MVPA was 44.30 min. Therefore, we plotted the effect of isochronous substitution on executive function from −40 min to 40 min between two behaviors in 5-min increments. The findings indicated that Stroop task z-scores exhibited a tendency to decrease when SP replaced SB, suggesting that the positive impact of sleep on inhibitory control in chronically ill older adults gradually increased. The substitution benefit was most significant when MVPA gradually replaced SB, with the difference value gradually increasing from 0 to −0.359 (95% CI = −0.656 to −0.061). It is noteworthy that the substitution benefit exhibited asymmetry when MVPA substituted for SB, suggesting that its detrimental effect on inhibitory control would progressively escalate as MVPA was progressively diminished and attained a maximum value of 1.165 (95% CI = 0.064 to 2.260) at −40. For the N-back task, z-scores exhibited a downward trend when LPA was substituted for SB, suggesting that a 5-min increase in LPA and a 5-min decrease in SB were associated with enhanced working memory. Conversely, a decrease in LPA accompanied by an increase in SB resulted in a consistent upward trend in standardized z-scores, which was associated with a decline in working memory performance.

## 4 Discussion

This cross-sectional study employed component data analysis to examine the relationship between 24-h activity behavior and executive function in older adults with chronic diseases, as well as their substitution effects. The findings of the study suggest a correlation between the increase in time spent in SP and MVPA among older adults suffering from chronic diseases and improved inhibitory control. A similar correlation was observed between the increase in time spent in LPA and improved working memory. The investigation revealed no substantial impact of physical activity on cognitive flexibility.

### 4.1 Analysis of 24-h activity in older adults with chronic diseases

Research findings indicate that among older adults with chronic diseases, SB accounts for nearly 50% of daily time, while LPA constitutes only 11.4%. In comparison with a study conducted in a healthy older adult population, participants in this study spent a higher proportion of their 24-h period in SB, while LPA accounted for only about half that amount (26). This finding suggests that older adults with chronic diseases may engage in less physical activity and spend a greater proportion of their day in SB. A substantial body of evidence has emerged that demonstrates a positive correlation between regular physical exercise and a reduced risk of developing chronic diseases. Given the fact that the present study concentrated on older adults suffering from chronic diseases, the investigated population may, by definition, manifest low levels of physical activity (27, 28). However, older adults with chronic diseases may encounter various constraints that hinder the achievement of recommended levels of physical activity. These constraints may include pain, limited opportunities for exercise, and mobility limitations (29). Consequently, these findings may offer a potential explanation for the observed prolonged sitting and reduced activity patterns among older adults with chronic diseases in this study. The variance matrix results indicate that MVPA exhibits low dependency on other activities (ln variance > 0.143), revealing its relative independence. This suggests that increasing MVPA may require active behavioral interventions rather than natural substitution, as it does not readily substitute for other activities. This may be related to the functional decline associated with chronic diseases (30, 31). Compared to LPA, MVPA places higher demands on the body’s energy supply system, cardiopulmonary function, and musculoskeletal system. Chronic conditions such as diabetes, cardiovascular disease, and bone disorders directly impair blood supply capacity, joint mobility, and energy availability, making it more challenging for older adults with chronic diseases to engage in MVPA (32, 33).

### 4.2 24-h activity and inhibitory control in older adults with chronic diseases

With respect to inhibitory control, the study found that increased minutes of MVPA in older adults with chronic diseases was associated with enhanced inhibitory control. Conversely, increased sedentary time was linked to weakened inhibitory function. In the subsequent isochronous substitution analysis, each unit increase in MVPA or sleep duration, accompanied by a corresponding reduction in sedentary time, was associated with improved inhibitory function. These findings suggest that increasing sleep or MVPA duration plays a crucial role in enhancing inhibitory function among older adults with chronic diseases, with MVPA demonstrating a more pronounced effect. This finding is consistent with the results of a questionnaire-based study assessing cognitive abilities (34). From a neurobiological perspective, the positive impact of MVPA on inhibitory function may occur through multiple pathways. The extant evidence suggests that regular physical activity of a moderate to vigorous intensity may have a protective effect on amyloid beta levels in the hippocampus and is associated with increased temporal lobe volume. This suggests that there may be a neuroanatomical basis for the promotion of inhibitory function by MVPA (10, 35). A study on older adults demonstrated that higher levels of MVPA were associated with greater prefrontal cortex oxygenation, which may further promote beneficial changes in cerebral vascular function, neurotrophic factors, and brain lactate metabolism, thereby enhancing executive function (36). Neuroimaging evidence suggests a correlation between age-related decline in inhibitory control and reduced activation in the prefrontal cortex and anterior cingulate cortex (37, 38). These findings suggest that MVPA enhances inhibitory control in older adults by increasing neural activity and physiological function in critical brain regions. Cardiovascular disease and bone disorders are two examples of chronic conditions that serve as risk factors for neurovascular unit dysfunction, homeostasis disruption, and neurohormonal response dysregulation in older adults. These factors have been demonstrated to induce neuronal damage in specific brain regions, expedite the accumulation of *β*-amyloid, and directly compromise the functionality of pertinent brain areas, culminating in a diminution of inhibitory control (39, 40). Therefore, for older adults with chronic diseases, gradually increasing MVPA can serve as a proactive compensatory strategy. This approach improves brain region structure and function to mitigate neurofunctional decline caused by chronic conditions, thereby counteracting the detrimental effects of sedentary behavior on inhibitory control.

Abnormal sleep quality or duration has been demonstrated to be closely associated with cognitive decline in older adults, and it is similarly influenced by prefrontal cortex function (41). Increasing sleep duration has been shown to promote immune system development in older adults, reduce the risk of chronic disease onset, and consequently alleviate protective brain region function while strengthening inhibitory control (11, 42). It is noteworthy that the findings of this study contrast with those of a cross-sectional study that examined the relationship between 24-h activity and executive function in community-dwelling older adults. This earlier study reported a significant positive effect of LPA on inhibitory control (26). This discrepancy may be attributed to the fundamentally different composition of activity patterns in the study populations. In this study, the proportion of LPA among older adults with chronic diseases was only half that observed in the comparison study. This substantial discrepancy in activity levels might have resulted in a threshold effect on health benefits, signifying that excessively low LPA levels may encounter challenges in producing discernible enhancements in inhibitory function.

### 4.3 24-h activity and working memory in older adults with chronic diseases

The present study found that increased sedentary time among older adults with chronic diseases was associated with poorer working memory performance, while increased LPA was associated with improved working memory. These findings are consistent with those of previous studies that employed n-back tasks (43, 44). However, the extant literature on the relationship between LPA and working memory has shown inconsistencies to date, which may be related to the outcome measures selected for n-back tasks (45). A body of research indicates that the capacity-limited working memory system deteriorates with aging. Concurrently, memory load increases with age, resulting in performance deficits. The 2-back task poses a greater challenge to memory storage in older adults compared to the 1-back task, potentially resulting in failure to accurately capture experimental outcomes and causing outcome bias (46).

The association between chronic diseases and working memory has been demonstrated in multiple studies. The extant literature suggests a correlation between impaired working memory performance and chronic conditions such as chronic low back pain, persistent inflammation, and type 2 diabetes (47–49). A study based on the UK Biobank found that participants with a higher proportion of sedentary time had a greater risk of non-communicable diseases compared to those with a lower proportion of sedentary time. These findings may elucidate the underlying mechanism for the elevated proportion of sedentary time observed in the present sample. A study that utilized an accelerometer as a measurement tool demonstrated that a reduction in sedentary time, coupled with an increase in LPA, promotes cognitive development in older adults and yields positive benefits for cerebrovascular health (50). This finding aligns with the partial substitution effect of physical activity observed in the present study. Given that the majority of participants (80%) had cardiovascular disease, it is plausible that underlying cardiovascular health benefits enhance the positive impact of LPA on working memory performance in older adults with chronic conditions.

The cognitive process of working memory is defined by the temporary storage and manipulation of information to achieve specific task objectives. At the level of physiological mechanisms, working memory has been extensively linked to the prefrontal cortex and the dorsolateral prefrontal cortex. Moreover, research conducted on animal models has demonstrated that hyperglycemia impairs activity in the hippocampus and anterior cingulate cortex (ACC) networks, thereby affecting working memory networks (51–54). Research has demonstrated that resistance band stretching exercises and low-intensity physical activity can enhance age-related brain atrophy patterns in older adults, leading to a reduction in age-related hippocampal volume loss and regional cortical atrophy. LPA is a more accessible form of physical activity, and it is easier for older adults with chronic diseases to adopt. It helps them reduce sedentary time and increase overall physical activity levels. For instance, a study combining fNIRS and ERP evidence demonstrated that Tai Chi intervention enhances neural activity levels and improves working memory in older adults (55). Consequently, the promotion of reduced sedentary behavior among older adults afflicted with chronic diseases through the augmentation of LPA has the potential to stimulate the activation of pertinent brain regions, thereby enhancing the efficacy of working memory performance.

### 4.4 24-h activity and cognitive flexibility in older adults with chronic diseases

With respect to cognitive flexibility, the present study found no significant association between physical activity behaviors and this domain. This result aligns with findings from assessments using the More-Odd-Shifting task among community-dwelling older adults (26). This discrepancy may be attributed to variations in assessment methodologies. Conventional cognitive flexibility assessments, such as the Trail Making Test, predominantly evaluate motor speed and visual attention. In contrast, the More-Odd-Shifting paradigm places greater emphasis on assessing pure cognitive switching ability. Such variations in measurement tools may compromise the comparability of research findings (56–58). Furthermore, factors such as variations in cognitive reserve among older adults afflicted with chronic diseases, as well as heterogeneity in the severity of the diseases, have the potential to influence the stability of research outcomes. Future studies should employ more consistent measurement tools, larger sample sizes, and longitudinal designs to further clarify this relationship.

In summary, for older adults with chronic diseases, increasing the proportion of LPA, MVPA, and sleep within their 24-h activity patterns can effectively improve inhibitory control and working memory dimensions of executive function. A comparison of this population with that of healthy older adults reveals that the former typically exhibits varying degrees of mobility limitations, pain, or fatigue. This, in turn, often results in a propensity to adopt sedentary lifestyles accompanied by suboptimal sleep quality (59). LPA is a more viable activity for older adults. It typically encompasses leisurely walks, housework, tai chi, square dancing, gardening, and similar pursuits. The enhancement of LPA participation has been demonstrated to not only augment opportunities for social engagement but also to mitigate the risk of cognitive impairment (60). For geriatric patients afflicted with chronic ailments, augmenting LPA signifies a more viable and secure enhancement approach. While increasing MVPA has been demonstrated to also benefit the development of executive function, certain chronic conditions (such as bone diseases, recent myocardial infarction, or uncontrolled hypertension) pose contraindications, making the elevation of MVPA considerably challenging. Given the observed disparity in the substitution benefits between MVPA and sedentary behavior, a strategy that involves reducing MVPA time to increase sedentary time is likely to result in substantial negative consequences. It is imperative for older adults afflicted with chronic diseases to sustain moderate increments in MVPA, while eschewing diminutions in MVPA duration. This strategy is instrumental in fostering the development of executive function in this demographic. Consequently, older adults afflicted with chronic conditions can enhance their daily lives by diversifying their physical activities, ensuring adequate sleep duration, and reducing sedentary behavior. This approach has been demonstrated to safeguard executive function levels and promote cognitive health.

## 5 Advantages and limitations

The present study employed objective measurement tools to investigate, for the first time, the relationship between 24-h physical activity behaviors and executive function in older adults with chronic diseases, as well as their substitution effects. The findings underscore the substantial positive impacts of SP, LPA, and MVPA on executive function. However, it is important to note that this study is not without its limitations. Errors in assessment may still occur during the 24-h behavioral evaluation process that utilizes accelerometers. For instance, activities such as bathing or swimming may be underreported. Future studies should endeavor to assess complete 24-h activity patterns. The cross-sectional design and limited sample size, which included a higher proportion of female participants, may limit the generalizability of the findings and restrict the scope of extrapolation. The present study was conducted among a chronic disease population and considered multiple chronic conditions. However, potential confounding factors between different conditions may have interfered with the experimental results, introducing potential bias in the inferences drawn. Future research should increase the sample size, collect data from different regions and chronic disease types, enhance the universality and representativeness of the findings, improve the wellbeing of older chronic disease populations, and provide factual evidence for developing more detailed and specific 24-h activity guidelines.

## 6 Conclusion

The following conclusions were reached by the study: it has been demonstrated that older adults afflicted with chronic diseases tend to lead sedentary lifestyles, which are typified by extended periods of sedentary behavior, such as prolonged sitting, and a marked reduction in physical activity over the course of a 24-h period. Secondly, the present study found that MVPA is associated with superior inhibitory control, whereas LPA is linked to enhanced working memory. Conversely, SB is associated with poorer inhibitory control and working memory. Thirdly, with regard to inhibitory control, an enhancement in SP or MVPA, as well as a reduction in SB, has been demonstrated to engender favorable outcomes, resulting in protracted enhancements over the course of time. A rise in LPA accompanied by a decline in SB has been shown to have a favorable impact on working memory. This impact is characterized by consistent enhancements that emerge as these activities supplant sedentary behavior over time. It is imperative for older adults afflicted with chronic diseases to prioritize the regulation of their 24-h time allocation, the reduction of prolonged sitting, the assurance of adequate sleep, and the maintenance of sufficient activity duration. These measures are crucial for the preservation of executive function performance and the promotion of cognitive health.

## Data Availability

The datasets presented in this article are not readily available because participants have requested that such data not be disclosed due to the inclusion of sensitive information, such as details of chronic diseases. Consequently, this dataset will not be released to the public. Requests to access the datasets should be directed to LW, wanglin123@126.com.
